# Light and dark sides of evidence-based and supportive ICU care for patients undergoing extracorporeal membrane oxygenation

**DOI:** 10.1186/s40560-023-00704-0

**Published:** 2023-12-07

**Authors:** Keibun Liu, Mohan Gurjar, Ricardo Kenji Nawa, Chi Ryang Chung, Kensuke Nakamura

**Affiliations:** 1 Non-Profit Organization ICU Collaboration Network (ICON), 2-15-13 Hongo, Bunkyo-Ku, Tokyo, 113-0033 Japan; 2 Critical Care Research Group, The Prince Charles Hospital, Brisbane, Australia; 3 Institute for Molecular Bioscience, The University of Queensland, Brisbane, Australia; 4 Department of Critical Care Medicine, Sanjay Gandhi Post Graduate Institute of Medical Sciences (SGPGIMS), Rae Bareli Road, Lucknow, India; 5 Hospital Israelita Albert Einstein, São Paulo, SP Brazil; 6 Department of Critical Care Medicine, Samsung Medical Center, Sungkyunkwan University School of Medicine, Seoul, Republic of Korea; 7 Department of Critical Care Medicine, Yokohama City University Hospital, 3-9, Fukuura, Kanazawa-Ku, Yokohama, Kanagawa 236-0004 Japan

**Keywords:** ABCDEF bundle, COVID-19, Extracorporeal membrane oxygenation, ICU liberation bundle, Pandemic

Dear Editor,

Battles against a critical illness never end even after survivorship, or rather the beginning of long-term hardship patients and families face to restore previous lives. The functional disabilities after ICU stay, or post-intensive care syndrome (PICS), is a physically, cognitively, and mentally devastating state that significantly reduces patient quality of life […] [1]. As prevention, evidence-based and supportive ICU care, such as the ‘ABCDEF’ bundle and nutrition therapy, is strongly recommended in daily clinical practice for all ICU patients, regardless of disease. PICS may get worse depending on the severity. Therefore, patients requiring extracorporeal membrane oxygenation (ECMO) to sustain their lives are at high risk of PICS development [2]. The benefits of ECMO have been well described and its cases have considerably increased [3]. However, the implementation of evidence-based and supportive ICU care for ECMO patients has not been adequately exposed.

We conducted a secondary analysis of previously published point prevalence studies on 3 dates (June 3, July 1, 2020, and January 27, 2021) with the aim of investigating the implementation of the ‘ABCDEF’ bundle and nutrition therapy (defined in Additional file 2: Table S1) for ECMO patients with mechanical ventilation (MV) patients as reference. A total of 60 ECMO patients and 778 MV patients were enrolled in 110 ICUs across 35 countries (Additional file 1: Figure S1 and Additional file 3: Table S2). Implementation of an entire ABCDEF bundle was extremely low in both groups. Compared to MV patients, ECMO patients, with longer ICU days, younger, and more use of renal replacement and vasopressors (Additional file 4: Table S3) demonstrated the higher implementation of Element ‘A’: pain assessments (75%), ‘C’: sedation assessments (90%), and ‘D’: delirium assessments (73%), while only one of the ten in the ECMO group received Element ‘B’: Spontaneous Awakening Trials and ‘E’: Early mobility and exercise, and one of the four received Element ‘F’: Family engagement and empowerment. More than half of both groups received 1500 kcal/day or 20 kcal/kg/day or more of energy, whereas protein provision of 1.2 g/kg/day or more was achieved for only 40%.

Large potentials to improve ICU care during ECMO were implied, though studies only captured performances on three days in the COVID-19 pandemic under high pressure on ICUs. The importance of pain, sedation, and delirium during ECMO might be relatively acknowledged, reflecting the higher requirements of analgesia and sedation than MV. However, we found an obvious opposite trend against the current recommendations of establishing whole-bundle care. Although no consensus on the optimal timings was set, light sedation, or ‘awake ECMO’, and early mobilization, which showed significant physiological benefits, were also rarely performed despite the relatively long stay in the ICU [4]. Considering high risk of new psychiatric symptoms after ECMO, family involvement should be essential [5]. Despite the synergy effect of nutrition therapy with the bundle, especially early mobility, only about half of patients with ECMO or less received a sufficient supply of protein.

In summary, large gaps between recommendations for ICU bundled-care and actual implementation were found. This is an urgent wake-up call to all ICU staff towards improving the quality of ICU care for patients requiring ECMO, the most severe and vulnerable patients, for a future of successful functional outcomes (Fig. 1).

**Fig. 1:**
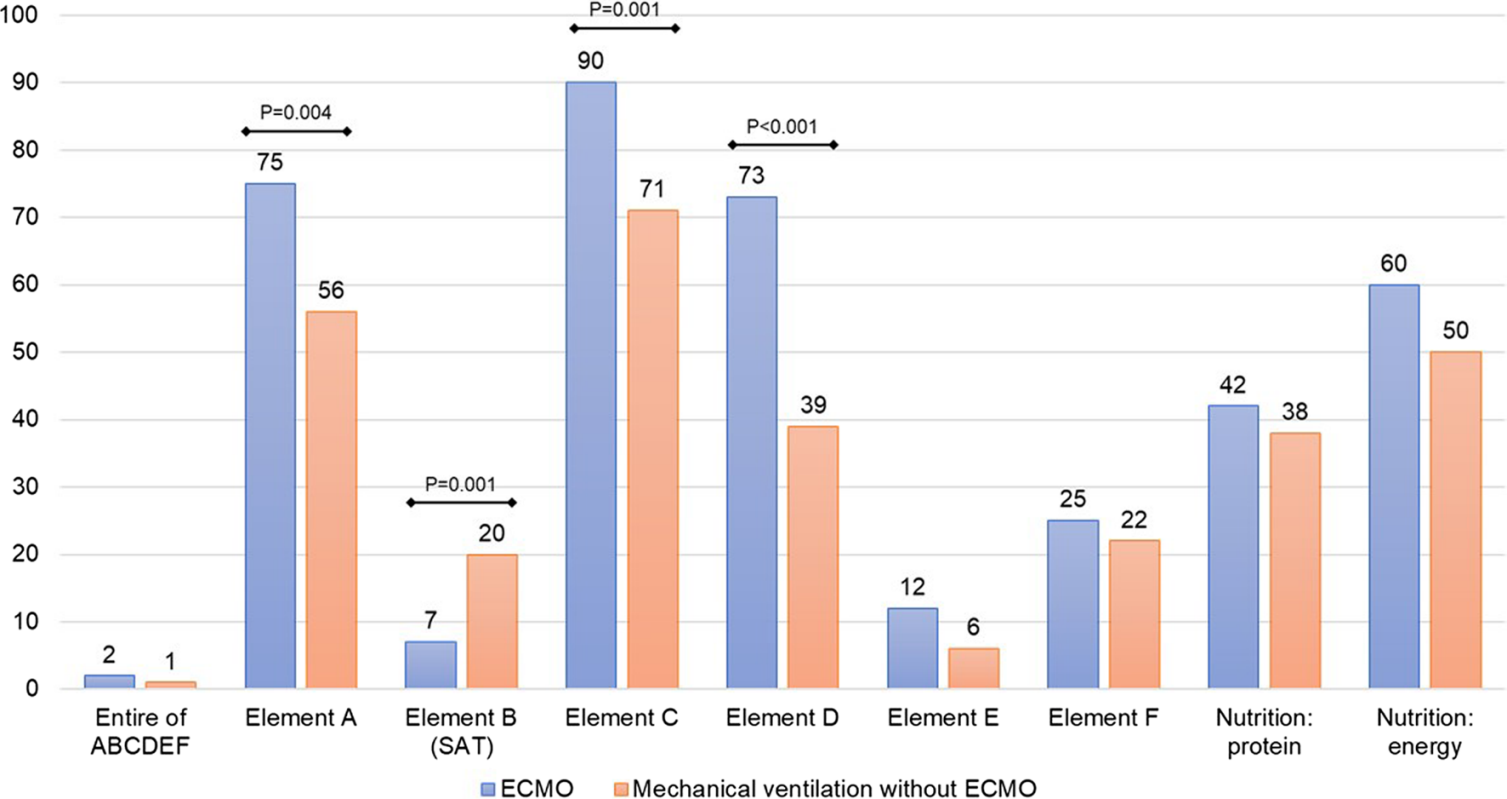
Implementation of the ABCDEF bundle and nutrition therapy for ICU patients. This figure indicates the implementation rates of the ABCDEF bundle and nutrition therapy for mechanically ventilated patients who were admitted to the ICUs on the survey dates without extracorporeal membrane oxygenation or with extracorporeal membrane oxygenation

### Supplementary Information

**Additional file 1: Figure S1.** Patient flow chart. All 57 ICUs participated in the survey on the 3rd of June, 2020, were included in the 73 ICUs participated in the survey on the 1st of July, 2020. The 73 ICUs participated in the survey on the 1st of July, 2020, and the 135 ICUs participated in the survey on the 27th of January, 2021, could be overlapped but not be able to be identified because of the anonymous nature of the survey response.

**Additional file 2: Table S1.** Definitions of the ‘ABCDEF’ bundle and nutrition therapy.

**Additional file 3: Table S2. **List of participating countries.

**Additional file 4: Table S3. **Baseline characteristics.

## Data Availability

Data set is available upon reasonable request.

## Abbreviations

ICU: Intensive care unit
PICS: Post-intensive care syndrome
ECMO: Extracorporeal membrane oxygenation
MV: Mechanical ventilation
SAT: Spontaneous awakening trial
