# Supplementary figures and images for: Light and dark sides of evidence-based and supportive ICU care for patients undergoing extracorporeal membrane oxygenation
Source: J Intensive Care. 2023 Dec 7;11:61. doi: 10.1186/s40560-023-00704-0 (PMC10701970; doi:10.1186/s40560-023-00704-0)

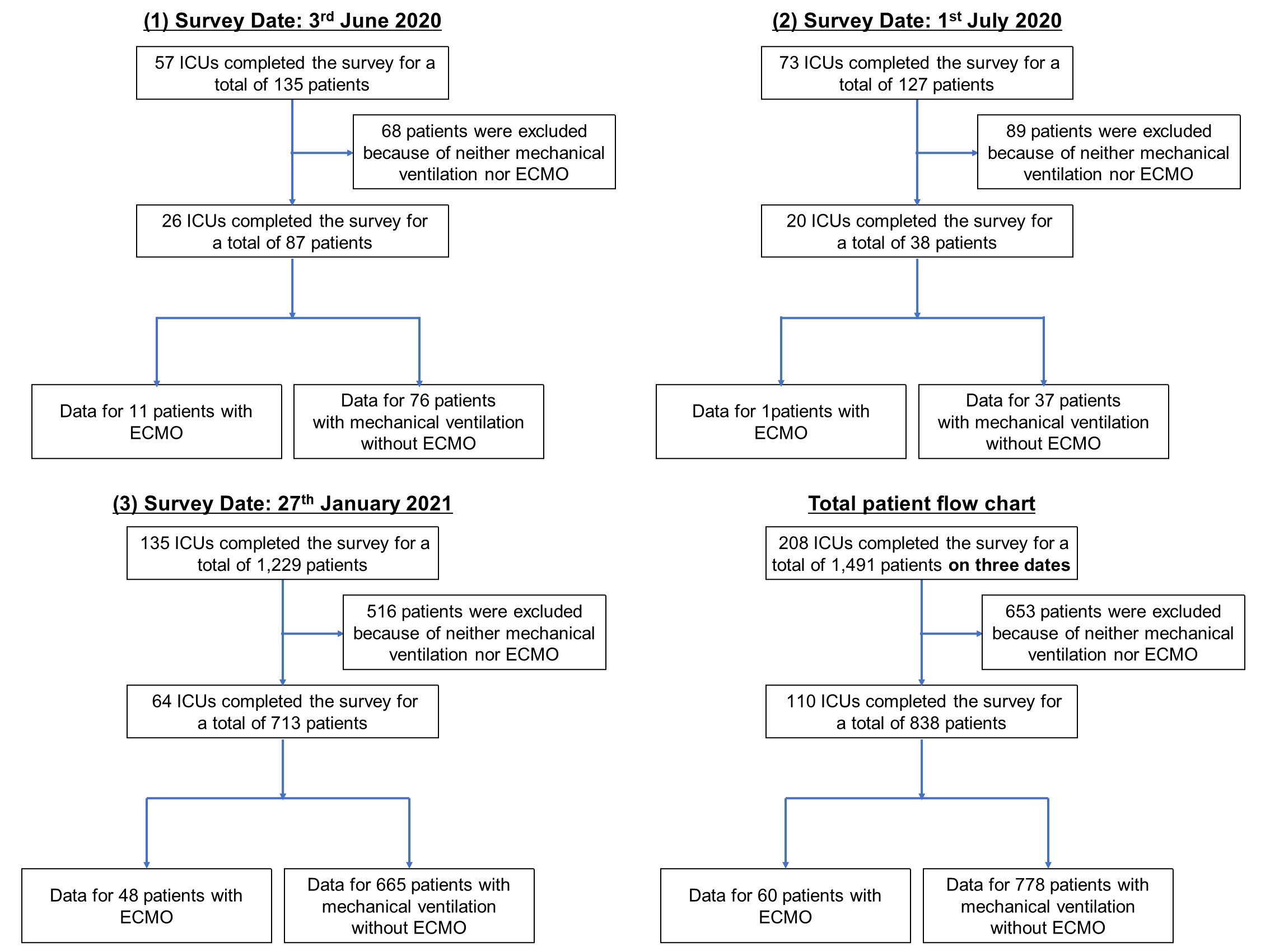

Supplement: Supplementary file 1 — Additional file 1: Figure S1. Patient flow chart. All 57 ICUs participated in the survey on the 3rd of June, 2020, were included in the 73 ICUs participated in the survey on the 1st of July, 2020. The 73 ICUs participated in the survey on the 1st of July, 2020, and the 135 ICUs participated in the survey on the 27th of January, 2021, could be overlapped but not be able to be identified because of the anonymous nature of the survey response. [file 40560_2023_704_MOESM1_ESM.jpg]
