# Supplementary material for: Light and dark sides of evidence-based and supportive ICU care for patients undergoing extracorporeal membrane oxygenation
Source: J Intensive Care. 2023 Dec 7;11:61. doi: 10.1186/s40560-023-00704-0 (PMC10701970; doi:10.1186/s40560-023-00704-0)
Supplement: Supplementary file 2 — Additional file 2: Table S1. Definitions of the ‘ABCDEF’ bundle and nutrition therapy. [file 40560_2023_704_MOESM2_ESM.docx]

**Supplementary e-Table1.** The definitions of the ‘ABCDEF’ bundle and nutrition therapy

| **ICU care** | **Operational definition** |
| --- | --- |
|  | |
| **Element A** | Regular standardized **pain** assessment using valid and reliable pain assessment scales 6 times or more per day. The pain assessment scales include NRS, CPOT, BPS, and others. |
| **Element B** | The **spontaneous awakening trials** is cessation of sedatives and narcotics or similar protocol to evaluate consciousness.  The **spontaneous breathing trials** were excluded from ‘Element B’ in this study since a majority of ECMO patients are not eligible for it. |
| **Element C** | Regular standardized **sedation** assessment using valid and reliable sedation assessment scales 6 times per day or more. The sedation assessment scales include RASS, SAS, Ramsay Sedation Scale, and others. |
| **Element D** | Regular standardized **delirium** assessment using valid and reliable delirium monitoring tools twice a day or more. The delirium assessment tools include CAM-ICU, ICDSC, and others. |
| **Element E** | **Early mobility and exercise** activities that were out of bed or higher. It is equal to a score of 4 or higher according to the IMS (i.e., dangling at edge of bed, standing at side of bed, walking to bedside chair, marching in place, walking in room or hall). |
| **Element F** | **Family engagement and empowerment** that a family member/significant other of this patient is educated on the ‘ABCDEF’ bundle and/or participate in at least one of the followings: rounds; conference; plan of care; or ‘ABCDEF’ bundle related care (e.g., re-orientation, calming talks, etc.). This element could be conducted in person or via online. |
| **Nutrition therapy** | |
| Total estimated energy (kcal/day or kcal/kg/day) | Total estimated nutritional energy (kcal), or the total energy provided to patients within the last 24 hours on the survey date, was 1,500 kcal/day or 20 kcal/kg/day or more. |
| Total estimated protein (g/kg/day) | Total estimated nutritional protein (g), or the total protein provided to patients within the last 24 hours on the survey date, was 1.2 g/kg/day or more. |

Abbreviations: BPS = behavioral pain scale, CAM-ICU = confusion assessment method for intensive care unit, CPOT = critical-care pain observation tool, ECMO = extracorporeal membrane oxygenation, g = gram, ICDSC = intensive care delirium screening checklist, ICU = intensive care unit, IMS = intensive care unit mobility scale, kcal = kilocalorie, kg = kilogram, NRS = numerical rating scale, RASS = Richmond agitation-sedation scale, SAS = sedation-agitation scale.

These definitions are based on the following references.

1. Liu K, Nakamura K, Katsukawa H, Nydahl P, Ely EW, Kudchadkar SR, et al. Implementation of the ABCDEF Bundle for Critically Ill ICU Patients During the COVID-19 Pandemic: A Multi-National 1-Day Point Prevalence Study. Frontiers in Medicine. 2021;8:2016.
2. Liu K, Nakamura K, Katsukawa H, Elhadi M, Nydahl P, Ely EW, et al. ABCDEF Bundle and Supportive ICU Practices for Patients With Coronavirus Disease 2019 Infection: An International Point Prevalence Study. Crit Care Explor. 2021;3:e0353.
