# Supplementary material for: Light and dark sides of evidence-based and supportive ICU care for patients undergoing extracorporeal membrane oxygenation
Source: J Intensive Care. 2023 Dec 7;11:61. doi: 10.1186/s40560-023-00704-0 (PMC10701970; doi:10.1186/s40560-023-00704-0)
Supplement: Supplementary file 3 — Additional file 3: Table S2. List of participating countries. [file 40560_2023_704_MOESM3_ESM.docx]

**Supplementary e-Table2.** **The list of participating countries**

| **Country** | **Participated survey number*** | | |
| --- | --- | --- | --- |
|  | Survery ‘1’  (June 3, 2020) | Survey ‘2’  (July 1, 2020) | Survey ‘3’  (January 27, 2021) |
| Argentina | X |  |  |
| Australia |  |  | X |
| Bangladesh |  |  | X |
| Brazil | X | X | X |
| Egypt | X | X | X |
| France | X |  | X |
| India | X |  | X |
| Iran | X |  | X |
| Iraq |  |  | X |
| Korea |  |  | X |
| Japan | X | X | X |
| Lebanon |  |  | X |
| Libya |  | X | X |
| Malaysia |  |  | X |
| Morocco |  |  | X |
| Namibia |  |  | X |
| Netherlands |  | X |  |
| Palestinian |  |  | X |
| Philippines | X | X |  |
| Poland |  |  | X |
| Portugal |  | X | X |
| Qatar |  |  | X |
| Russian Federation |  | X |  |
| Saudi Arabia | X |  | X |
| Singapore |  |  | X |
| South Africa |  |  | X |
| Spain |  | X | X |
| Sudan | X |  |  |
| Syria |  |  | X |
| Switzerland | X |  |  |
| Turkey | X | X |  |
| United Kingdom | X |  | X |
| United States | X | X |  |
| Venezuela |  |  | X |
| Yemen |  | X |  |
| *A total of 35 countries participated in all three surveys | | | |
