# Supplementary material for: Light and dark sides of evidence-based and supportive ICU care for patients undergoing extracorporeal membrane oxygenation
Source: J Intensive Care. 2023 Dec 7;11:61. doi: 10.1186/s40560-023-00704-0 (PMC10701970; doi:10.1186/s40560-023-00704-0)
Supplement: Supplementary file 4 — Additional file 4: Table S3. Baseline characteristics. [file 40560_2023_704_MOESM4_ESM.docx]

**Supplementary e-Table3. Baseline characteristics**

| **Variable** | **ECMO**  **(*n* = 60)** | **MV without ECMO**  **(*n* = 778)** |
| --- | --- | --- |
| COVID-19 infection, no., (%) | 30 (50) | 371 (48) |
| Age, *years*, no., (%) |  |  |
| < 20 | 2 (3) | 28 (4) |
| ≧20 to 50 | 15 (25) | 136 (17) |
| ≧50 to 60 | 14 (23) | 144 (19) |
| ≧60 to 70 | 18 (30) | 223 (29) |
| ≧70 to 80 | 9 (15) | 176 (23) |
| ≧ 80 | 2 (3) | 64 (8) |
| Gender, *male*, no., (%) | 43 (72) | 530 (68) |
| BMI, *kg/m^2^*, no., (%) |  |  |
| < 18.5 | 2 (3) | 55 (7) |
| ≧ 18.5 to 25 | 17 (28) | 263 (34) |
| ≧ 25 to 30 | 20 (33) | 253 (33) |
| ≧ 30 to 35 | 13 (22) | 126 (16) |
| ≧ 35 | 8 (13) | 81 (10) |
| ICU length of stay, *days*, median [IQR] | 12 [4-15] | 8 [4-16] |
| RRT support, no., (%) | 16 (27) | 101 (13) |
| Patients receiving continuous vasopressor, no., (%) | 31 (52) | 361 (46) |

Data in table are presented as median [IQR] or number (%).

BMI = body mass index, COVID-19 = coronavirus disease 2019, ECMO = extracorporeal membrane oxygenation, ICU = intensive care unit, IQR = interquartile range, MV = mechanical ventilation, RRT = renal replacement therapy.
